# Use of Reverse Shock Index Multiplied by Simplified Motor Score in a Five-Level Triage System: Identifying Trauma in Adult Patients at a High Risk of Mortality

**DOI:** 10.3390/medicina60040647

**Published:** 2024-04-18

**Authors:** Po-Chen Lin, Meng-Yu Wu, Da-Sen Chien, Jui-Yuan Chung, Chi-Yuan Liu, I-Shiang Tzeng, Yueh-Tseng Hou, Yu-Long Chen, Giou-Teng Yiang

**Affiliations:** 1 Department of Emergency Medicine, Taipei Tzu Chi Hospital, Buddhist Tzu Chi Medical Foundation, New Taipei 231, Taiwan; taipeitzuchier@gmail.com (P.-C.L.); skyshangrila@gmail.com (M.-Y.W.); brianann75@gmail.com (Y.-T.H.); yulong0129@gmail.com (Y.-L.C.); 2 Department of Emergency Medicine, School of Medicine, Tzu Chi University, Hualien 970, Taiwan; 3 Graduate Institute of Injury Prevention and Control, Taipei Medical University, Taipei 110, Taiwan; 4 Department of Emergency Medicine, Cathay General Hospital, Taipei 106, Taiwan; 5 School of Medicine, Fu Jen Catholic University, Taipei 242, Taiwan; 6 School of Medicine, National Tsing Hua University, Hsinchu 300, Taiwan; 7 Department of Orthopedic Surgery, Taipei Tzu Chi Hospital, Buddhist Tzu Chi Medical Foundation, New Taipei 231, Taiwan; 8 Department of Orthopedics, School of Medicine, Tzu Chi University, Hualien 970, Taiwan; 9 Department of Research, Taipei Tzu Chi Hospital, Buddhist Tzu Chi Medical Foundation, New Taipei 970, Taiwan; istzeng@gmail.com

**Keywords:** trauma, reverse-shock index multiplied by simplified motor score, Taiwan Triage and Acuity Scale, triage

## Abstract

*Background and Objectives*: The Taiwan Triage and Acuity Scale (TTAS) is reliable for triaging patients in emergency departments in Taiwan; however, most triage decisions are still based on chief complaints. The reverse-shock index (SI) multiplied by the simplified motor score (rSI-sMS) is a more comprehensive approach to triage that combines the SI and a modified consciousness assessment. We investigated the combination of the TTAS and rSI-sMS for triage compared with either parameter alone as well as the SI and modified SI. *Materials and Methods*: We analyzed 13,144 patients with trauma from the Taipei Tzu Chi Trauma Database. We investigated the prioritization performance of the TTAS, rSI-sMS, and their combination. A subgroup analysis was performed to evaluate the trends in all clinical outcomes for different rSI-sMS values. The sensitivity and specificity of rSI-sMS were investigated at a cutoff value of 4 (based on previous study and the highest score of the Youden Index) in predicting injury severity clinical outcomes under the TTAS system were also investigated. *Results*: Compared with patients in triage level III, those in triage levels I and II had higher odds ratios for major injury (as indicated by revised trauma score < 7 and injury severity score [ISS] ≥ 16), intensive care unit (ICU) admission, prolonged ICU stay (≥14 days), prolonged hospital stay (≥30 days), and mortality. In all three triage levels, the rSI-sMS < 4 group had severe injury and worse outcomes than the rSI-sMS ≥ 4 group. The TTAS and rSI-sMS had higher area under the receiver operating characteristic curves (AUROCs) for mortality, ICU admission, prolonged ICU stay, and prolonged hospital stay than the SI and modified SI. The combination of the TTAS and rSI-sMS had the highest AUROC for all clinical outcomes. The prediction performance of rSI-sMS < 4 for major injury (ISS ≥ 16) exhibited 81.49% specificity in triage levels I and II and 87.6% specificity in triage level III. The specificity for mortality was 79.2% in triage levels I and II and 87.4% in triage level III. *Conclusions*: The combination of rSI-sMS and the TTAS yielded superior prioritization performance to TTAS alone. The integration of rSI-sMS and TTAS effectively enhances the efficiency and accuracy of identifying trauma patients at a high risk of mortality.

## 1. Introduction

Traumatic injury is a major global health problem, contributing to both mortality and disability and placing a substantial burden on healthcare systems [1]. Several triage tools have been developed for use in both prehospital and hospital settings, including the Canadian Triage and Acuity Scale (CTAS) [2], the Emergency Severity Index (ESI) [3], the Manchester Triage Scale [4], the Taiwan Triage and Acuity Scale (TTAS) [5,6], and the Australasian Triage Scale [7]. The ESI is a five-level triage scale based on physical signs and expected resource use, which focuses on quickly categorizing patients in settings with limited resources. One advantage of the ESI is the rapid identification of patients needing immediate attention [8]. However, multiple studies have highlighted an overrepresentation of ESI III assignments and a lack of accurate differentiation in patient acuity levels. This trend has been linked to emergency department (ED) overcrowding and poorer patient outcomes. Addressing these issues is crucial for enhancing ED efficiency and ensuring optimal patient care [9,10]. The ATS is based on adult physiological predictors (airway, breathing, circulation, and disability). A meta-analysis included six studies showed that the pooled coefficient for the ATS was substantial at 0.428 (95%CI 0.340–0.509) and the mis-triage rate was less than fifty percent. Compared to ESI, which has a strong tendency towards categorizing patients as level 2, ATS can appropriately distribute patients in triage levels [11,12]. In addition, the Manchester Triage Scale is a five-level triage algorithm that consists of 52 flowcharts, covering patients’ chief signs and symptoms. Each flowchart in turn consists of additional signs and symptoms, named discriminators, which are ranked by priority [13]. Compared to ESI in the ED triage, the mean length of stay by using MTS triage was significantly lower [14]. CTAS and TTSA are based on presenting signs and symptoms, which provides more information regarding early treatment than the ESI (which is a triage tool that predicts ED resource allocation). In the study by Joany M Zachariasse et al. [15], the authors included 66 eligible studies and evaluated 33 different triage systems, revealing numerous different triage systems are being used; they found that many lack a rigorous evaluation. The most commonly used and evaluated triage systems, CTAS, ESI, and MTS, show a moderate–good validity in identifying high- and low-urgency patients. In the results, there is no strong evidence supporting differences between CTAS, ESI, and MTS; therefore, none of them should be preferred over the other.

Accurate triage is essential in ensuring that patients with trauma receive the appropriate level of care, given that undertriage or overtriage can lead to poor outcomes and waste valuable resources [16]. The TTAS is reliable for triaging patients in emergency departments (EDs) in Taiwan [5,6]. TTAS can vary based on regional healthcare systems. Different regions or countries may use different three-tier emergency classification systems, or they may be customized based on local medical needs and resources. Therefore, the specific implementation of TTAS systems may vary by region. The TTAS was adapted from the CTAS and maintains most of the key features of the CTAS. In addition, it uses a patient classification system that prioritizes treatment on the basis of five levels of acuity—level I (most urgent) to level V (least urgent) [17]—and is based on chief complaints and first-order modifiers (such as vital signs (including respiration, hemodynamics, consciousness level, and body temperature), pain severity, and injury mechanism (for patients with trauma) to determine triage severity. Although the TTAS system uses explicit threshold levels for hemodynamic stability (e.g., tachycardia [140 bpm]/bradycardia [50 bpm], with or without symptoms of shock or blood pressure < 70 mm Hg) as order modifiers, most triage decisions are still based on chief complaints. According to an analysis of 36,395 major patients with trauma from the Nationwide Emergency Department Sample of the United States, nearly one in three patients who experienced major trauma were undertriaged [18]. Similarly, relying solely on the TTAS system for trauma triage may result in patients being undertriaged or overtriaged. Triage systems must be frequently evaluated to assess their efficacy in identifying patients at high risk of severe injury in order to ensure high patient safety and appropriate and timely utilization of ED resources.

The 2021 National Guidelines for the Field Triage of Injured Patients (field triage guideline) updated and added new criteria for field triage [19]. The two major concepts were the application of the shock index (SI) and simplified consciousness assessment. These two components can be combined in a novel tool known as the reverse SI (rSI) multiplied by simplified motor score (sMS), as rSI-sMS, which aims to provide a relatively user-friendly, easily applicable, and comprehensive approach to triage. The decision to utilize rSI multiplied by sMS rather than SI multiplied by sMS is grounded in the correlation of lower values of rSI or sMS with poorer outcomes. Conversely, higher values of SI would suggest a worse outcome. This reasoning remains consistent when employing rSI multiplied by GCS as well [20]. This novel tool incorporates the rSI and the 3-point sMS to assess both hypovolemic shock status and neurological condition in patients with trauma.

In this study, we evaluated the effectiveness of incorporating rSI-sMS to identify patients with severe injury at high risk of mortality within a level category of the TTAS system. We hypothesized that the addition of rSI-sMS would decrease the proportion of undertriage and overtriage among patients with trauma.

## 2. Methods

### 2.1. Study Design and Cohort

The present retrospective cohort analysis used data from the Taipei Tzu Chi Hospital registry and was approved by the Institutional Review Board of Taipei Tzu Chi Hospital (IRB number: 11-XD-148 and 12-XD-079). This hospital’s trauma database includes hospitalized patients with ICD-9-CM codes 800–959 (excluding 905–909 and 930–939) or ICD-10-CM codes S00–T98 (excluding T15–T19 and T90–T98). The ICD-9-CM codes 905–909 (ICD-10-CM codes T90–T98) pertain to the late effects of injury, poisoning, toxic effects, and other external causes, rather than the immediate effects associated with acute trauma. As they are not directly related to acute trauma, they were excluded from this study. Furthermore, the ICD-9-CM codes 930–939 (ICD-10-CM codes T15–T19) represent the effects of foreign bodies entering the body through natural orifices, which are also unrelated to trauma. Consequently, they were also excluded from our analysis. In addition, the database records 152 variables associated with trauma injury, including demographics, injury mechanism, injury types, injury severity, vital signs, surgical intervention, and in-hospital mortality. We included all patients with major traumatic injuries from January 2009 to June 2019; we excluded those under the age of 20 years because of differences in normal vital sign ranges for pediatric patients.

### 2.2. TTAS System

The TTAS is a computerized decision support system used in EDs in Taiwan and was adapted from the CTAS [5,21,22]. The TTAS classifies patients into three domains: trauma (14 categories and 41 chief complaints), nontrauma (13 categories and 125 chief complaints), and environmental injuries (11 chief complaints). Triage severity is assessed on the basis of chief complaints and first-order modifiers, including vital signs, consciousness level, and pain severity. When first-order modifiers are insufficient in determining the appropriate triage acuity level, second-order modifiers—such as visual disturbance for eye trauma or neurologic deficit for head, neck, and back trauma—are applied. Patients are prioritized on the basis of their acuity level as follows: level 1, resuscitation; level 2, emergency; level 3, urgent; level 4, less urgent; and level 5, nonurgent.

In Taipei Tzu Chi Hospital, to qualify as a candidate for the role of triage nurse in the ED, a nurse must meet the following requirements: (1) completion of 3 years of basic nursing training, including general nursing, observation nursing, emergency and critical care nursing, pediatric nursing, trauma nursing, and care for toxic and environmental injuries; (2) recognition by the Taiwan Nurses Association based on the Clinical Ladder System for the nurses program in a hospital; and (3) completion of triage classification education courses. The candidates are led by senior triage nurses to perform triage classification for a minimum of 100 patients for 5 days. After passing the evaluation, a nurse is qualified as a formal triage nurse. Currently, Taipei Tzu Chi Hospital has 25 emergency triage nurses.

One designated triage nurse who has undergone specific training regarding the application of the five-level TTAS protocol and the computer-assisted system generates the TTAS level for each patient in real time. Studies have demonstrated that the TTAS is a reliable triage system that accurately prioritizes treatment, avoids overtriage, and efficiently allocates appropriate resources to ED patients.

### 2.3. Variable Measurements

We retrieved data regarding the analyzed patients’ clinicodemographic characteristics, including age, sex, chronic diseases, triage level, vital signs, and injury mechanism and severity. Vital sign data—including those related to heart rate, systolic blood pressure (SBP), diastolic blood pressure (DBP), respiratory rate, Glasgow Coma Scale (GCS) score, and sMS—were recorded upon arrival at the hospital and employed to calculate rSI-sMS. The sMS assesses the GCS motor response, with a score of 2 indicating that the patient can follow commands (equivalent to a GCS motor subscale score of 6), a score of 1 indicating that the patient can localize to pain (equivalent to a GCS motor subscale score of 5), and a score of 0 indicating that the patient has a GCS motor subscale score of ≤4. However, the rSI-sMS was calculated as 1/SI × sMS. We cannot use a score of 0 of sMS to calculate rSI-sMS. Therefore, we modified the sMS by changing the highest score to 3 and the lowest score to 1. We also included the SI and mSI for analysis. The SI was calculated as heart rate (HR)/SBP; the mSI was calculated as HR/mean arterial pressure. Based on a previous study [23], we used 4 as the cutoff value of rSI-sMS for predicting trauma patients with high risk of mortality.

We used the injury severity score (ISS) to determine trauma severity. Major trauma was defined as an ISS score of ≥16. Patients with traumatic brain injury (TBI) were stratified into mixed TBI (head abbreviated injury scale (AIS) score > 3 and any other AIS score ≥ 0) and isolated TBI (head AIS score > 3 and any other AIS score = 0) groups. We defined the geriatric population as age ≥ 65 years for subgroup analysis.

### 2.4. Outcomes

The primary outcome was in-hospital mortality. The secondary outcomes were admission to the intensive care unit (ICU), readmission to the ICU, ICU length of stay (LOS), prolonged ICU stay (>14 days), total hospital stay, and prolonged hospital stay (>30 days).

### 2.5. Statistical Analysis

Demographic data, injury data, and clinical outcomes were analyzed using SPSS (Version 20.0, SPSS, Chicago, IL, USA). In this paper, we assessed the normality of continuous data using the Kolmogorov–Smirnov test. Each continuous variable is reported as the mean ± standard deviation for normally distributed data and the median with interquartile range for nonnormally distributed data. Categorical variables are reported as numbers and percentages. Independent-samples t-tests were conducted for normally distributed continuous variables, whereas Mann-Whitney U tests were performed for nonnormally distributed continuous variables. Pearson’s chi-squared test or Fisher’s exact test was used for categorical variables. Multivariable logistic regression was performed to predict the primary and secondary outcomes in patients with trauma, with significant variables or variables deemed important included in the analysis. The area under the receiver operating characteristic curve (AUROC) was calculated to assess the discrimination of the logistic regression model for each outcome. A *p* value of <0.05 was considered statistically significant, and all tests were two-sided.

## 3. Results

### 3.1. Patient Characteristics

Of the 13,144 eligible patients, 1384 were excluded owing to their age being <20 years (*n* = 1198), death upon arrival (*n* = 151), or insufficient data for calculating rSI-sMS (*n* = 35). Ultimately, we included the data of 11,760 patients for analysis; these patients’ clinicodemographic characteristics are presented in Table 1. Triage level II had the largest proportion of patients (51.7%), followed by level III (41.8%) and then level I (6%). Compared with triage levels II and III, the level I population had higher proportions of men (63.9%) and older adults (age ≥ 65 years; 38.4%); lower SBP, DBP, and GCS scores; a higher HR and respiratory rate; and a higher proportion of isolated brain injury (39.5%). Nonpenetrating injuries were the primary cause of trauma in this study, with traffic accidents and falls being the most common mechanisms. Among all the triage levels, level I had the highest incidence of road transport injuries (46.8%), whereas level III had the highest incidence of low falls (46.1%). Cardiovascular disease was the most common underlying condition across all the triage levels. The triage level III population had higher proportions of cardiovascular disease and diabetes mellitus than the level I and II populations.

The level I group had a higher ISS than the other groups, with 42.2% of patients presenting with an ISS of ≥16. The revised trauma score (RTS) was lowest for triage level I, with 49.6% of the triage level I patients with RTS < 7. The level I group had a higher proportion of patients requiring admission to the ICU, readmission, and prolonged ICU stay. The triage level III group had the highest operation rate (72.1%), whereas the level I group had the highest reoperation rate (10.5%), the highest complication rate (24.8%), the longest total LOS (19.8%), and the highest in-hospital mortality rate (15.0%).

### 3.2. Prioritization Performance of Patients Using the TTAS System

**Figure 1:**
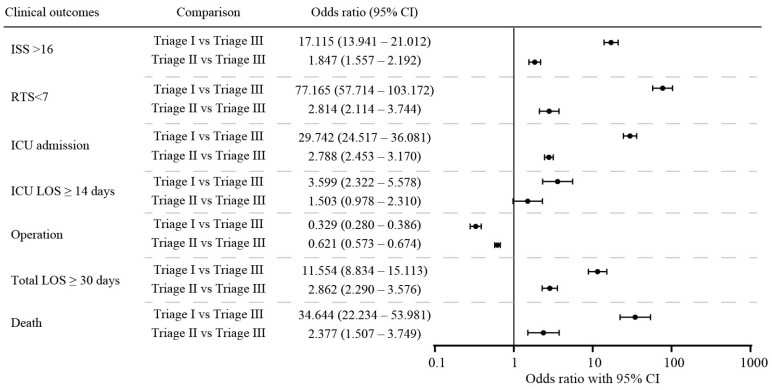
Odds ratios for logistic regression analysis for injury severity and clinical outcomes between different triage levels in the TTAS.

We observed that the TTAS system was effective in prioritizing patients with major trauma, with significant differences observed between the triage level groups related to RTS < 7, ISS ≥ 16, hospital stay, ICU stay, the proportion of patients admitted to the ICU, prolonged ICU stay (≥14 days), prolonged hospital stay (≥30 days), and mortality. Specifically, the level I and II triage groups had higher odds ratios for major injury (as indicated by RTS < 7 and ISS ≥ 16), ICU admission, prolonged ICU stay, prolonged hospital stay, and mortality and lower odds ratios for operation than the level III group (Figure 1).

### 3.3. Risk Stratification Based on rSI-sMS < 4 in the TTAS

The results of risk stratification based on rSI-sMS < 4 in the TTAS are presented in Table 2. The triage level I group had the highest percentage of patients (59.8%) with rSI-sMS < 4, followed by the level II group (17.6%) and then the level III (12.7%) group. In all three triage levels, a significant difference regarding injury severity (ISS ≥ 16 and RTS < 7) was noted between patients with rSI-sMS < 4 and those with rSI-sMS ≥ 4. The rSI-sMS < 4 group had a higher RTS and ISS, a higher proportion of ISS ≥ 16 and RTS < 7 patients, and less favorable outcomes related to ICU admission, reoperation, complications, prolonged hospitalization, and mortality than the rSI-sMS ≥ 4 group. Notably, no significant differences in clinical outcomes were observed between the two groups in triage levels IV and V.

**Figure 2:**
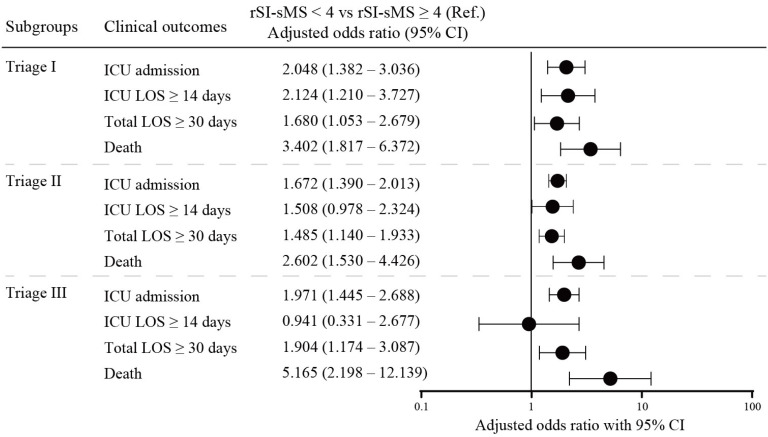
Odds ratios for logistic regression analysis for clinical outcomes of the rSI-sMS < 4 group at different triage levels.

**Figure 3:**
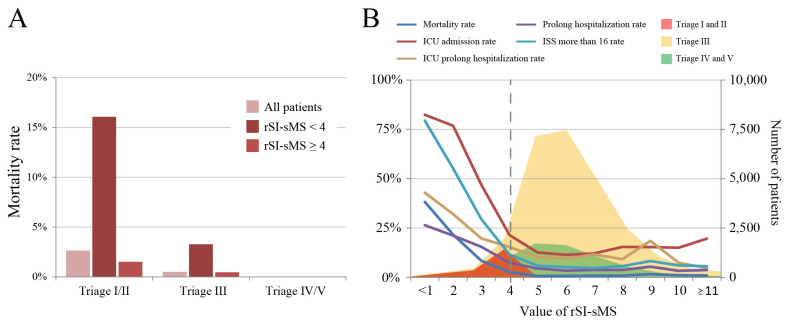
(**A**) Mortality rate of the rSI-sMS < 4 group in different triage levels. (**B**) Trends in all clinical outcomes for different rSI-sMS values.

Table 3 compares the results of the SI, mSI, and rSI-sMS AUROCs for predicting trauma outcomes. The TTAS and rSI-sMS had higher AUROCs for mortality, ICU admission, prolonged ICU stay, and prolonged hospital stay than the SI and mSI. The combination of the TTAS and rSI-sMS had a higher AUROC for all clinical outcomes than that of the TTAS and the SI or mSI. The subgroup analysis of the rSI-sMS < 4 group across multiple triage levels is presented in Figure 2. In triage levels I–III, patients with rSI-sMS < 4 had higher odds ratios of ICU admission, prolonged hospital stay, and mortality than those with rSI-sMS ≥ 4. Additionally, in triage levels I and II, patients with rSI-sMS < 4 had a higher risk of prolonged ICU stay than those with rSI-sMS ≥ 4. The mortality rates of the rSI-sMS < 4 group across the various triage levels are presented in Figure 3A. The overall mortality rates were 3% in triage levels I and II and 1% in triage level III. Patients with rSI-sMS < 4 had a significantly higher mortality rate than those with rSI-sMS ≥ 4 in triage levels I and II (16.07% vs. 1.52%, respectively) and level III (3.28% vs. 0.47%, respectively).

The trends for all clinical outcomes for the various rSI-sMS values are presented in Figure 3B. A cutoff value of 4 for rSI-sMS facilitated effective discrimination between all the clinical outcomes (Table 4). The trend of the mortality rate, prolonged hospitalization rate, ICU admission rate, ICU prolonged hospitalization rate, and major injury rate (ISS ≥ 16) remained relatively flat after the application of the cutoff value. The predictive performance of rSI-sMS < 4 for major injury was 81.49% specificity in triage levels I and II and 87.6% specificity in triage level III. The sensitivity and specificity for mortality were 64.4% and 79.2%, respectively, in triage levels I and II and 36% and 87.4%, respectively, in triage level III.

## 4. Discussion

Triage is an essential element of the trauma care system used to determine the extent of injuries and treatment priorities to facilitate appropriate resource allocation to patients with trauma. However, accurately identifying patients who require trauma care can be challenging given that prehospital providers and emergency physicians often have limited data to inform such identification. The rSI-sMS method was promoted based on the current concept of the Field Triage Guidelines in 2021 [19]. The rSI-sMS method has two major advantages: use of the SI to reflect patients’ hemodynamic status, and use of the sMS instead of the GCS to simplify consciousness assessment in order to reflect neurological status. The present study demonstrated that rSI-sMS is effective in identifying high-risk populations, including those with major injury (ISS ≥ 16), high mortality and ICU admission rates, and prolonged hospitalization and ICU stays. Although the five-level TTAS can effectively prioritize patients with major trauma, patients with rSI-sMS < 4 had less favorable outcomes than those with rSI-sMS ≥ 4 within the same triage level. This finding implies that rSI-sMS should be included in the triage system for better identification of patients who require trauma care and more accurate prediction of their outcomes to enhance the quality of care and improve trauma outcomes.

The TTAS system mainly categorizes and determines triage levels on the basis of patients’ chief complaints, which are subdivided into 163 categories. Although vital signs are included as modifiers in the primary adjusting variables, chief complaints still play a major role in determining triage levels. Therefore, the determination of chief complaints during triage is crucial. In cases of a lack of triage nurses or triage training resources, inaccuracies may arise in the determination of triage levels. In addition, nurses may develop their own personalized usage of the TTAS through years of practice, which could lead to variations in the interpretation and integration of the tool, resulting in lower interrater reliability. A study that analyzed 100 patients arriving by ambulance assessed by five experienced ED nurses reported that the overall interrater agreement of the CTAS was moderate (global Kappa coefficient: 0.44) [24], suggesting a need for further research to verify the reliability of the CTAS. The use of an objective scoring tool can mitigate the limitations of the TTAS and reduce differences among triage personnel. The rSI-sMS method is suitable for quick use by emergency medical workers upon patient arrival without any additional equipment or cost. The core variables used in the calculation of rSI-sMS are those that are commonly collected in clinical practice, and this calculation is based on a simple algorithm. An rSI-sMS score of <4 can be used as a primary trigger for action in an ED. In addition, using rSI-sMS < 4 as a risk stratification tool in the TTAS can facilitate the identification of high-risk patients. In the current study, we observed that even after risk stratification using the TTAS, patients with rSI-sMS < 4 in triage levels I–III had greater injury severity and less favorable clinical outcomes, as reflected by their relatively high odds ratios for mortality, ICU admission, prolonged total LOS, and prolonged ICU stay. Overall, implementing rSI-sMS < 4 as a risk stratification tool in the TTAS can improve patient outcomes and help to optimize resource utilization. In addition, we expect that morbidity and mortality can decrease with the addition of rSI-sMS to the TTAS system; however, further prospective studies are necessary to verify this assumption.

In the present study, rSI-sMS was significantly associated with trauma outcomes and enhanced the ability of the TTAS to predict mortality, ICU admission, prolonged total LOS, and ICU stay in patients with trauma. These findings are in line with those of other studies that have suggested that incorporating vital sign prediction scoring systems into the triage process can help reduce delays in evaluating and treating undertriaged patients and can decrease the associated morbidity [25]. Jung-Fang Chuang et al. [25] used the rSI as an additional criterion under the TTAS had better classification performance in triage levels I and II. In addition, patients with a severe SI of <1 also had worse outcomes. Furthermore, the subgroup analysis revealed that the combination of rSI-sMS and triage had better predictive performance than the combination of SI and mSI [16]. Another concern is inaccurate prioritization in levels II and III of the TTAS system when patients have an rSI of <1. In the present study, the prioritization performance of rSI-sMS in levels II and III of the TTAS system did not yield the same results as Chuang et al. [25]. This difference may have been partially due to our cohort having higher severe injury and higher mortality rates: 51.7% of all the analyzed patients were classified as level II and 41.8% were classified as level III. Although the TTAS exhibited strong prioritization performance, using rSI-sMS as an additional criterion may increase physicians’ attention to high-risk patients with trauma.

This study had several strengths. First, we investigated a novel prediction scoring system that had better predictive performance than the SI and mSI. We applied an objective scoring system, namely rSI-sMS, in the TTAS for the reclassification of high-risk patients with trauma to mitigate the limitations of the TTAS. Second, we analyzed an Asian triage system, namely the TTAS, which has not been widely evaluated. The TTAS is typically designed to screen all patients evenly on the basis of chief complaints. Although the main description-based injury assessment in the TTAS is quick and easy, it overlooks the risk of misalignment due to ignorance of personal reactions, such as those related to age and comorbidities. Therefore, the incorporation of an objective additional criterion is useful for increasing predictive performance. Finally, the present large cohort was adjusted for many confounders to eliminate the possibility of any potential influence and thus reflect real-world conditions.

Notably, this study also had some limitations. First, the retrospective design precluded the collection of data related to vital signs; consequently, we excluded patients with missing records for vital signs of interest. However, patients with missing records for age, sex, and other physiological variables were not excluded. The number of cases with missing values was found to be negligible (<0.3%); thus, imputation was deemed unnecessary. Imputing data from vital sign records would have been inappropriate and could have introduced inaccuracies into the findings. Second, detailed triage information was not included in the database. We recognize that such information could further validate the performance of TTAS prioritization and further our understanding of the heterogeneity of triage determination. Accordingly, future studies are recommended to include such data to enhance the accuracy and comprehensiveness of their findings. Third, we did not include injured patients who died before arrival at the hospital, and this omission may have introduced bias. Moreover, this study lacked access to long-term outcome data after discharge and follow-up information from other hospitals, both of which could have facilitated more comprehensive understanding of the performance of the TTAS with rSI-sMS prioritization. Finally, although the Tzu Chi Trauma Database includes data related to a large number of patients over the preceding decade, this study was conducted at a single center; thus, the findings may have limited generalizability. Accordingly, further prospective multicenter studies are warranted to validate our results.

## 5. Conclusions

In this study, patients with rSI-sMS < 4 had more severe injuries (ISS ≥ 16) and experienced worse outcomes—including prolonged hospital and ICU stay, a higher proportion of ICU admission, and increased in-hospital mortality—compared with patients with rSI-sMS ≥ 4. Although the five-level TTAS system was effective in prioritizing patients with major trauma, patients with rSI-sMS < 4 had less favorable outcomes than those with rSI-sMS ≥ 4 within the same triage level (I–III). The combination of rSI-sMS and the TTAS yielded superior prioritization performance to rSI-sMS, the TTAS alone, the SI, or the mSI. Thus, our findings indicate that incorporating rSI-sMS into the TTAS system can help identify patients with serious injuries who may require reclassification to a higher triage level.

## Figures and Tables

**Table 1:** Clinicodemographic characteristics of the included patients, stratified by triage level.

Characteristics	Triage I	Triage II	Triage III	Triage IV/V	*p*-Value
Patient number	711 (6.0%)	6080 (51.7%)	4914 (41.8%)	55 (0.5%)	
Age (years)	56 (39–74)	59 (43–77)	63 (47–77)	52 (36–62)	<0.001
Age < 65 ys	438 (61.6%)	3562 (58.6%)	2642 (53.8%)	43 (78.2%)	<0.001
Age ≥ 65 ys	273 (38.4%)	2518 (41.4%)	2272 (46.2%)	12 (21.8%)	
Sex, *n* (%)					<0.001
Female	257 (36.1%)	2772 (45.6%)	2577 (52.4%)	25 (45.5%)	
Male	454 (63.9%)	3308 (54.4%)	2337 (47.6%)	30 (54.5%)	
Vital sign					
SBP	143 (115–170)	147 (127–169)	148 (129–165)	141 (122–154)	<0.001
DBP	81.5 (68–96)	85 (74–97)	85 (76–95)	81 (73–93)	<0.001
HR	88 (76–105)	84 (74–96)	82 (72–93)	85 (72–97)	<0.001
RR	19 (18–20)	18 (18–20)	18 (18–20)	18 (18–20)	<0.001
GCS score	14 (7–15)	15 (15–15)	15 (15–15)	15 (15–15)	
Injury score systems					
rSIsms	3.5 (2.0–5.0)	5.2 (4.3–6.3)	5.4 (4.5–6.3)	4.8 (4.1–5.8)	<0.001
rSIsms < 4	425 (59.8%)	1069 (17.6%)	625 (12.7%)	13 (23.6%)	<0.001
rSIsms ≥ 4	286 (40.2%)	5011 (82.4%)	4289 (87.3%)	42 (76.4%)	
Injury severity					
RTS	7.1 (6.0–7.8)	7.8 (7.8–7.8)	7.8 (7.8–7.8)	7.8 (7.8–7.8)	<0.001
RTS < 7	353 (49.6%)	211 (3.5%)	62 (1.3%)	0 (0.0%)	<0.001
ISS	11 (9–20)	9 (4–9)	9 (4–9)	4 (4–9)	<0.001
ISS ≥ 16	300 (42.2%)	444 (7.3%)	201 (4.1%)	2 (3.6%)	<0.001
Isolated head injury *	281 (39.5%)	1080 (17.8%)	500 (10.2%)	10 (18.2%)	<0.001
Injury type					<0.001
Penetration	57 (8.0%)	366 (6.0%)	142 (2.9%)	1 (1.8%)	
Non-penetration	654 (92.0%)	5714 (94.0%)	4772 (97.1%)	54 (98.2%)	
Mechanism of injury					<0.001
Road transport	333 (46.8%)	2255 (37.1%)	1691 (34.4%)	20 (36.4%)	
Low fall	142 (20.0%)	2298 (37.8%)	2264 (46.1%)	19 (34.5%)	
High fall	129 (18.1%)	789 (13.0%)	556 (11.3%)	5 (9.1%)	
Others	107 (15.0%)	738 (12.1%)	403 (8.2%)	11 (20.0%)	
Comorbidity					
CNS diseases	48 (6.8%)	405 (6.7%)	283 (5.8%)	0 (0.0%)	0.052
CVD	142 (20.0%)	1791 (29.5%)	1626 (33.1%)	9 (16.4%)	<0.001
Respiratory diseases	13 (1.8%)	155 (2.5%)	109 (2.2%)	1 (1.8%)	0.510
CKD	19 (2.7%)	206 (3.4%)	150 (3.1%)	1 (1.8%)	0.568
Diabetes mellitus	63 (8.9%)	770 (12.7%)	683 (13.9%)	3 (5.5%)	0.001
ICU care					
ICU admission	491 (69.1%)	1052 (17.3%)	343 (7.0%)	5 (9.1%)	<0.001
Re-admission ICU	8 (1.1%)	27 (0.4%)	9 (0.2%)	0 (0.0%)	0.001
ICU LOS, days	6 (3–13)	4 (3–7)	4 (3–6)	2 (2–3)	<0.001
LOS < 14 days	372 (75.8%)	928 (88.2%)	315 (91.8%)	5 (100.0%)	<0.001
LOS ≥ 14 days	119 (24.2%)	124 (11.8%)	28 (8.2%)	0 (0.0%)	
Surgical intervention					
Operation	327 (46.0%)	3748 (61.6%)	3544 (72.1%)	29 (52.7%)	<0.001
Re-operation	75 (10.5%)	188 (3.1%)	81 (1.6%)	0 (0.0%)	<0.001
Complications	176 (24.8%)	830 (13.7%)	165 (3.4%)	2 (3.6%)	<0.001
Total LOS	11 (5–25)	6 (4–10)	6 (4–8)	5 (3–7)	<0.001
<30 days	570 (80.2%)	5279 (94.2%)	4811 (97.9%)	54 (98.2%)	<0.001
≥30 days	141 (19.8%)	351 (5.8%)	103 (2.1%)	1 (1.8%)	

CKD: chronic kidney disease; CVD: cardiovascular diseases; ISS: injury severity score; RTS: revised trauma score; LOS: length of stay; ICU: intensive care unit. * Isolated head injury: patients with an AIS code limited to the head and no AIS-coded injury in any other region.

**Table 2:** Risk stratification between patients with rSI-sMS < 4 and ≥ 4 in different triage levels.

Outcomes	Triage I		Triage II		Triage III		Triage IV/V	
	rSI-sMS < 4	rSI-sMS ≥ 4	rSI-sMS < 4	rSI-sMS ≥ 4	rSI-sMS < 4	rSI-sMS ≥ 4	rSI-sMS < 4	rSI-sMS ≥ 4
Patient number	425 (59.8%)	286 (40.2%)	1069 (17.6%)	5011 (82.4%)	625 (12.7%)	4289 (87.3%)	13 (23.6%)	42 (76.4%)
Injury severity								
ISS ≥ 16	241 (56.7%) ***	59 (20.6%) ***	134 (12.5%) ***	310 (6.2%) ***	40 (6.4%) **	161 (3.8%) **	0 (0.0%)	2 (4.8%)
RTS < 7	315 (74.1%) ***	38 (13.3%) ***	147 (13.8%) ***	64 (1.3%) ***	39 (6.2%) ***	23 (0.5%) ***	0 (0.0%)	0 (0.0%)
ICU care								
ICU admission	334 (78.6%) ***	157 (54.9%) ***	267 (25.0%) ***	785 (15.7%) ***	70 (11.2%) ***	273 (6.4%) ***	1 (7.7%)	4 (9.5%)
Re-admission ICU	7 (1.6%)	1 (0.3%)	9 (0.8%) *	18 (0.4%) *	3 (0.5%)	6 (0.1%)	0 (0.0%)	0 (0.0%)
ICU LOS ≥ 14 days	98 (29.3%) ***	21 (13.4%) ***	39 (14.6%)	85 (10.8%)	5 (7.1%)	23 (8.4%)	0 (0.0%)	0 (0.0%)
Surgical intervention								
Operation	211 (49.6%) *	116 (40.6%) *	640 (59.9%)	3108 (62.0%)	425 (68.0%) *	3119 (72.7%) *	6 (46.2%)	23 (54.8%)
Re-operation	57 (13.4%) **	18 (6.3%) **	54 (5.1%) ***	134 (2.7%) ***	18 (2.9%) **	63 (1.5%) **	0 (0.0%)	0 (0.0%)
Complications	121 (28.5%) **	55 (19.2%) **	197 (18.4%) ***	633 (12.6%) ***	40 (6.4%) ***	125 (2.9%) ***	0 (0.0%)	2 (4.8%)
Total LOS ≥ 30 days	106 (24.9%) ***	35 (12.2%) ***	89 (8.3%) ***	262 (5.2%) ***	24 (3.8%) ***	79 (1.8%) ***	0 (0.0%)	1 (2.4%)
Death	91 (21.4%) ***	16 (5.6%) ***	25 (2.3%) ***	48 (1.0%) ***	9 (1.4%) ***	16 (0.4%) ***	0 (0.0%)	0 (0.0%)

* *p* < 0.05, ** *p* < 0.01, *** *p* < 0.001.

**Table 3:** Comparison of SI, mSI, and rSI-sMS AUROC curves for predicting mortality, ICU admission, prolonged ICU stay (≥14 days), and prolonged hospital stay (≥30 days).

Scoring Systems	Mortality		ICU Admission		Prolong ICU Stay		Prolong Total Hospital Stay	
	AUROC (95% CI)	*p*-Value	AUROC (95% CI)	*p*-Value	AUROC (95% CI)	*p*-Value	AUROC (95% CI)	*p*-Value
SI	0.505 (0.458–0.552)	0.819	0.522 (0.506–0.537)	0.003	0.527 (0.488–0.567)	0.147	0.547 (0.522–0.573)	<0.001
mSI	0.541 (0.495–0.587)	0.046	0.535 (0.519–0.550)	<0.001	0.545 (0.506–0.584)	0.018	0.559 (0.534–0.584)	<0.001
rSI-sMS	0.733 (0.688–0.778)	<0.001	0.605 (0.590–0.621)	<0.001	0.623 (0.584–0.662)	<0.001	0.616 (0.591–0.642)	<0.001
Triage	0.780 (0.743–0.816)	<0.001	0.702 (0.689–0.715)	<0.001	0.621 (0.584–0.657)	<0.001	0.676 (0.654–0.699)	<0.001
Triage + SI	0.770 (0.733–0.807)	<0.001	0.699 (0.686–0.713)	<0.001	0.617 (0.579–0.655)	<0.001	0.689 (0.667–0.711)	<0.001
Triage + mSI	0.780 (0.744–0.816)	<0.001	0.703 (0.690–0.717)	<0.001	0.623 (0.585–0.660)	<0.001	0.692 (0.670–0.713)	<0.001
Triage + rSI-sMS	0.797 (0.759–0.835)	<0.001	0.714 (0.701–0.728)	<0.001	0.641 (0.603–0.679)	<0.001	0.699 (0.677–0.721)	<0.001

**Table 4:** Performance of the rSI-sMS score for clinical outcomes at different triage levels.

Outcomes.	Triage I/II				Triage III				Triage IV/V			
	Sens.	Spec.	PLR	NLR	Sens.	Spec.	PLR	NLR	Sens.	Spec.	PLR	NLR
ISS ≥ 16	50.40%	81.49%	2.72	0.61	19.9%	87.6%	1.60	0.91	----	----	----	----
ICU admission	39.0%	83.0%	2.29	0.73	20.4%	87.9%	1.68	0.91	20.0%	76.0%	0.83	1.05
ICU LOS ≥ 14 days	56.4%	64.3%	1.58	0.68	17.9%	79.4%	0.86	1.03	----	----	----	----
Operation	20.9%	76.3%	0.88	1.04	12.0%	85.4%	0.82	1.03	20.7%	73.1%	0.77	1.08
Total LOS ≥ 30 days	39.6%	79.4%	1.92	0.76	23.3%	87.5%	1.86	0.88	----	----	----	----
Death	64.4%	79.2%	3.10	0.45	36.0%	87.4%	2.86	0.73	----	----	----	----

Sens., sensitivity; Spec., specificity; PLR, positive likelihood ratio; NLR, negative likelihood ratio.

## Data Availability

The data is unavailable due to privacy andor ethical restrictions.
